# Diet, physical activity and screen time but not body mass index are associated with the gut microbiome of a diverse cohort of college students living in university housing: a cross-sectional study

**DOI:** 10.1186/s12866-018-1362-x

**Published:** 2018-12-12

**Authors:** Corrie M. Whisner, Juan Maldonado, Brandon Dente, Rosa Krajmalnik-Brown, Meg Bruening

**Affiliations:** 1 College of Health Solutions, Arizona State University, Phoenix, AZ USA; 2 Center for Fundamental and Applied Microbiomics, Biodesign Institute, Arizona State University, Tempe, AZ USA; 3 ASU Genomics Core, Biodesign Institute, Arizona State University, Tempe, AZ USA; 4 Swette Center for Environmental Biotechnology, Biodesign Institute, Arizona State University, Tempe, AZ USA; 5 School of Sustainable Engineering and the Built Environment, Fulton Schools of Engineering, Arizona State University, Tempe, AZ USA

**Keywords:** Microbiome, Microbiota, Adolescence, Pediatric, Obesity, Physical activity, Diet, Lifestyle behaviors

## Abstract

**Background:**

Modifiable lifestyle factors (e.g. dietary intake and physical activity) are important contributors to weight gain during college. The purpose of this study was to evaluate whether associations exist between body mass index, physical activity, screen time, dietary consumption (fat, protein, carbohydrates, and fiber), and gut microbial diversity during the first year of college. Racially/ethnically diverse college students (*n* = 82; 61.0% non-white) at a large Southwestern university completed self-reported physical activity and 24-h recall dietary assessments, height and weight measurements, and provided one fecal sample for gut microbiome analysis. Fecal microbial community composition was assessed with Illumina MiSeq next-generation sequencing of PCR amplified 16S rRNA genes. Post-hoc analyses compared microbial diversity by groups of high and low physical activity and fiber intake using QIIME and LEfSe bioinformatics software.

**Results:**

No statistically significant differences were observed between body mass index and gut microbiome abundance and diversity. Median daily consumption of dietary fiber was 11.2 (7.6, 14.9) g/d, while the median self-reported moderate-to-vigorous physical activity (MVPA) was 55.7 (27.9, 79.3) min/d and screen time 195.0 (195.0, 315.0) min/d. Microbial analysis by LEfSe identified *Paraprevotellaceae*, *Lachnospiraceae,* and *Lachnospira* as important phylotypes in college students reporting greater MVPA, while *Enterobacteriaceae* and *Enterobacteriales* were more enriched among students reporting less MVPA (*p* < 0.05). *Barnesiellaceae*, *Alphaproteobacteria*, and *Ruminococcus* were more abundant taxa among those consuming less than the median fiber intake (*p* < 0.05). Post-hoc analyses comparing weighted UniFrac distance metrics based on combined categories of high and low MVPA and fiber revealed that clustering distances between members of the high MVPA-low fiber group were significantly smaller when compared to distances between members of all other MVPA-fiber groups (*p* < 0.0001).

**Conclusions:**

Habitual fiber consumption and MVPA behaviors help explain the differential abundance of specific microbial taxa and overall gut microbial diversity differences in first-year college students.

**Electronic supplementary material:**

The online version of this article (10.1186/s12866-018-1362-x) contains supplementary material, which is available to authorized users.

## Background

Obesity remains a persistent public health issue affecting 78.6 million adults in the United States [1]. The transition from high school to college known as emergent adulthood is a vulnerable period of life which is frequently characterized by weight gain [2–6], making it an important period for prevention and intervention [5, 7]. This transition period is usually marked by leaving home for the first time, a new environment, building new friendships and social networks, and greater independence in overall decision making [2]. The incidence of obesity during this transition period is reportedly high and is thought to persist throughout adulthood [8, 9]. Obesity is influenced by various factors including the environment [10], dietary intake [11], physical activity [12], and the intestinal microbiome [13, 14]. Higher incidence of obesity increases the risk of cardiometabolic conditions including hypertension, dyslipidemia, type 2 diabetes mellitus, heart disease, and cancer [9, 15].

Recent research suggests possible links between modifiable lifestyle factors, the gut microbiome, and health outcomes including obesity [16, 17]. Findings for physical activity among college students are bleak, with most studies suggesting a lack of physical activity among this population [6, 18–20]. First-year students specifically, have reported that the transition to college makes it difficult to maintain health and physical activity due to lack of intrinsic motivation, loss of routine, and fewer opportunities for organized sports participation [21]. Decreases in physical activity have been associated with changes in body composition, including increased fat mass and decreased lean body mass [22]. This suggests that changes in body composition may be a sensitive indicator of lifestyle changes during the freshman year.

Dietary behaviors adopted by college students frequently include meals at ‘all-you-can-eat’ facilities, evening snacking, junk food consumption, and dieting; these behaviors have all been associated with weight gain [23]. Changes in dietary behaviors ultimately result in greater consumption of energy-dense, nutrient-poor foods such as sugar-sweetened beverages, fried foods, and salty snacks [6, 24]. Ultimately these foods displace nutrient-rich fruits, vegetables and whole grains containing dietary fiber which promote the development of a healthy gut microbiome. College students habitually have inadequate (~ 18 g) dietary fiber consumption [25].

Studies show that dietary alterations can rapidly modify the gut microbiome [26]. Switching mice from a low-fat, plant-based diet to a high-fat/high-sugar diet negatively impacted the gut microbiome composition [26, 27]. A comparison of children from Western Europe and Burkina Faso (BF) revealed significant differences in the gut microbiome composition with high dietary fiber consumption among BF children resulting in greater *Bacteroidetes* relative to *Firmicutes* [28]. While components of plant-based diets have been reviewed and are thought to increase gut microbiome diversity via their dietary fiber composition [29], the influence of physical activity on the gut microbiome is an emerging area of research with very few human studies, as recently reviewed [30].

Gut microbiota composition differences have been observed between active and sedentary women with health-associated microbes *Akkermansia muciniphila*, *Fecalibacterium prausnitzii*, *Bifidobacteria longum*, and *Roseburia hominis* being more abundant in women meeting daily physical activity recommendations [31]. *Faecalibacterium* and *Roseburia* were also abundant in type-1 diabetics and healthy controls matched for high physical fitness with no differences by health status [32]. Specifically, aerobic exercise training for 6 weeks appears to shift the gut microbial composition and function as greater similarities were observed between obese and lean individuals irrespective of dietary intake [33]. Conversely, a study among elite rugby athletes found that both protein consumption and physical activity increased microbial diversity when compared to obese and normal weight controls [34]. One other study among women with breast cancer suggested that fitness level did not significantly associate with the relative abundance of specific gut microbes [35].

The gut during childhood and adolescence exhibits greater interpersonal variation and lower bacterial diversity compared to adults [36, 37]. This reduced diversity appears to create a more plastic and malleable gut microbiome [36, 37] which may fuel growth and allow greater and lasting microbial shifts in response to physical activity and diet. University students living on campus generally experience major changes in lifestyle habits, including physical activity and diet [29, 38]. Given that behaviors established during the college years may persist throughout adulthood and increase the risk of obesity and cardiometabolic diseases, this population provides a unique opportunity to expand our understanding of the role of physical activity and diet on the gut microbiome. The objective of this cross-sectional, observational study was to characterize the gut microbiome of a racially/ethnically diverse cohort of college students living in the dorms and assess possible associations with body mass index, measures of physical activity (moderate-to-vigorous physical activity), sedentary behavior (screen time), and dietary (fat, protein, carbohydrates, and fiber) intake.

## Results

### Participant characteristics

A total of 82 participants (57.3% female; 31.7% Hispanic) provided a fecal sample, MVPA and screen time data (Table 1). A subsample (*n* = 68; 60.3% female; 57.3% non-white) also provided 24-h dietary recall data. The median (IQR) percentage of kilocalories consumed from protein, fat and carbohydrate were 16.2 (14.2, 18.8) %, 35.8 (30.0, 40.8) %, and 47.9 (39.7, 54.4) %, respectively (Table 1). Both protein and carbohydrate consumption were within the acceptable macronutrient distribution range (AMDR) of 10–35% and 45–65%, respectively; while the median fat consumption fell slightly outside the AMDR range of 20–35% [39]. The median (IQR) self-reported daily intake of sugar consumed was 65.5 (47.6, 104.6) g/d. Median daily consumption of dietary fiber for males (*n* = 27) and females (*n* = 41) was 8.7 (7.1, 14.2) g/d and 11.4 (8.6, 16.8) g/d, respectively, for which both fell below the AMDR for males (38 g/d) and females (25–26 g/d) [39]. Self-reported MVPA (*p* = 0.133) and screen time (*p* = 0.441) did not differ by BMI classification. Self-reported screen time and MVPA were not significantly correlated (Spearman rho = − 0.143, *p* = 0.199).

**Table 1:** Sociodemographic and key variables of college students living in residence halls (*n* = 82)

Variable	
Age (years) *mean ± SD*	18.4 ± 0.6
Sex *% (n)*	
Male	42.7 (35)
Female	57.3 (47)
Residence hall *% (n)*	
A	37.8 (31)
B	62.2 (51)
Race/ethnicity *% (n)*	
Hispanic	31.7 (26)
White	39.0 (32)
Other	29.3 (24)
Body Mass Index (kg/m^2^) *mean ± SD*	24.4 ± 5.5
< 18.5 kg/m^2^*% (n)*	6.1 (5)
18.5–24.9 kg/m^2^*% (n)*	57.3 (47)
25.0–29.9 kg/m^2^*% (n)*	22.0 (18)
≥ 30.0 kg/m^2^*% (n)*	14.6 (12)
Diet^a^*median (IQR)*	
Carbohydrates (g)	165.7 (125.1, 240.7)
Fiber (g)	11.2 (7.6, 14.9)
Protein (g)	61.8 (42.4, 85.4)
Fat (g)	63.3 (38.8, 84.8)
Moderate-to-vigorous physical activity (min/day) *median (IQR)*	55.7 (27.9, 79.3)
Screen time (min/day) *median (IQR)*	195.0 (195.0, 315.0)

^a^Sample size decreases to *n* = 68 for diet data due to missing 24-h dietary recalls; *IQR*, interquartile range; *SD*, standard deviation

### Gut microbiota and behaviors

Amplicon high-throughput sequencing resulted in an average of 60,000 16S rRNA gene amplicon reads per sample. Rarefaction curves based on observed species, Chao1 and Faith’s PD (phylogenetic diversity) metrics suggested that adequate sampling depth was at 17,768 sequences. The median *Firmicutes:Bacteroidetes* ratio was 0.65 (0.39, 1.23). This ratio did not differ by BMI group (*p* = 0.413) or median categories of dietary protein (*p* = 0.763), fat (*p* = 0.469), carbohydrate (*p* = 0.683), and fiber (*p* = 0.835) intake. Similarly, the F:B ratios between MVPA (*p* = 0.583) and screen time (*p* = 0.323) categories did not differ significantly.

**Fig. 1:**
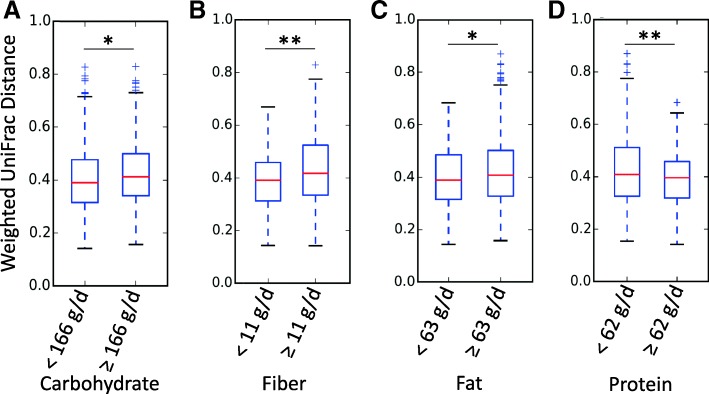
Distance metrics by (**a**) carbohydrate, (**b**) fiber, (**c**) fat, and (**d**) protein consumption groups. Groups were created by separating self-reported values that fell above and below median daily intakes. Dietary intake was obtained from automated self-report 24-h dietary recalls. Significant differences in distance metrics between members of one group compared to another are denoted as *p < 0.05, **p < 0.001

Chao1, observed OTU, and PD whole tree alpha-diversity did not differ significantly by categories (above or below the median) of dietary fat, protein, carbohydrate, or fiber. While no obvious visual differences in beta diversity (between-sample) were observed via PCoA plots (Additional file 1), comparison of distributions of distances showed significant within-group differences for carbohydrate, fiber and protein using both unweighted and weighted UniFrac data (weighted data are shown in Fig. 1). For fat consumption, only the within-group weighted UniFrac distances were significantly different between high and low-fat. Despite these differences in beta-dispersion, PERMANOVA results indicated that between group differences in weighted and unweighted distance metrics were not significantly influenced by dietary factors.

**Fig. 2:**
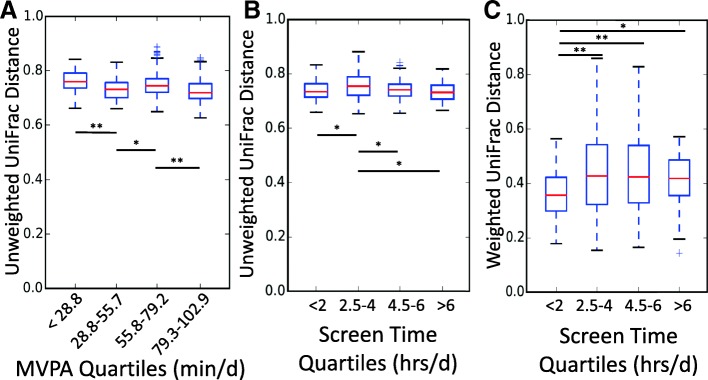
Differences in distance metrics by self-reported (**a**) MVPA and (**b** and **c**) screen time. MVPA and screentime were self-reported using validated survey questions. Significant differences in distance metrics between members of one group compared to another group are denoted as *p < 0.05; **p < 0.0001. MVPA, moderate-to-vigorous physical activity

Chao1, observed OTUs, and PD whole tree alpha-diversity metrics did not differ significantly by categories of MVPA or screen time, suggesting that physical activity and sedentary behaviors were not associated with species richness or evenness in this cohort of college students. No obvious visual differences in beta diversity (between-sample) were observed via PCoA plots (Additional file 1) when evaluating MVPA and screen time quartiles. Upon comparison of UniFrac distance metrics with Bonferroni corrections, significant differences in beta diversity were observed between MVPA groups when evaluating the median unweighted but not weighted within-group UniFrac distances (Fig. 2a). As unweighted UniFrac distances help to explain the presence of less abundant, rather than most abundant taxa; this result suggests that less abundant taxa may differ by self-reported physical activity levels. Both unweighted and weighted UniFrac within-group distances differed by self-reported total daily sedentary time or time spent in front of a screen (Fig. 2b and c). These data suggest that presence of both less and most abundant taxa may be characteristic of students with less (2.5–4 h) sedentary time compared to all other groups. Despite within-group differences in distances, no significant between-group differences in weighted and unweighted distances were observed for MVPA and sedentary time, as analyzed by PERMANOVA.

**Fig. 3:**
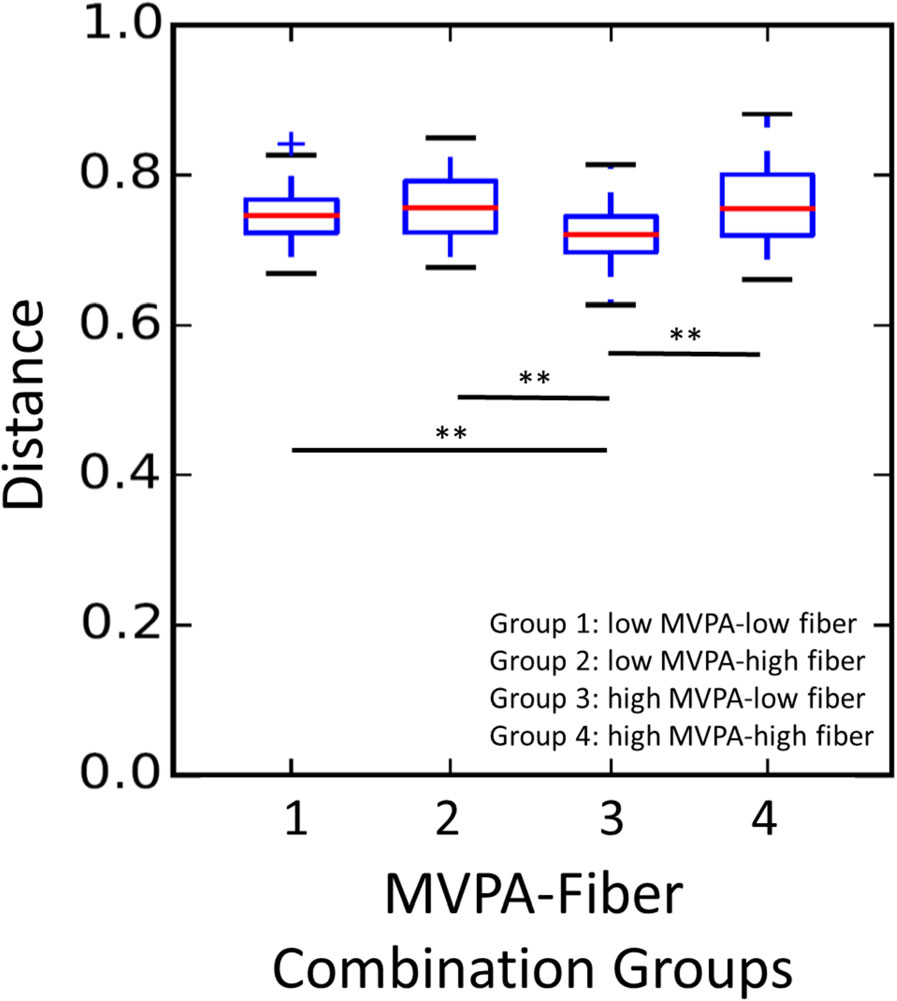
Beta diversity (unweighted UniFrac data) by categories of combined high and low MVPA and fiber. Median MVPA (55.7 min/d) and dietary fiber (11 g/d) consumption were used to create high and low groupings. Significant differences in distance metrics between members of one group compared to another group are denoted as **p < 0.0001. MVPA, moderate-to-vigorous physical activity

To further explore the influence of MVPA and dietary fiber consumption, in post-hoc analyses, we examined the beta-dispersion (weighted and unweighted UniFrac distances) within groups of participants characterized by merged MVPA and fiber categories (Group 1: low MVPA-low fiber, Group 2: low MVPA-high fiber, Group 3: high MVPA-low fiber, and Group 4: high MVPA-high fiber). Unweighted UniFrac data suggested that the high MVPA-low fiber group (Group 3) had significantly shorter distances among group members when compared to within-group distances of all other MVPA-fiber combination groups (Fig. 3, *p* < 0.001). When distances were partitioned by group and compared via PERMANOVA, no significant between-group differences were observed.

**Fig. 4:**
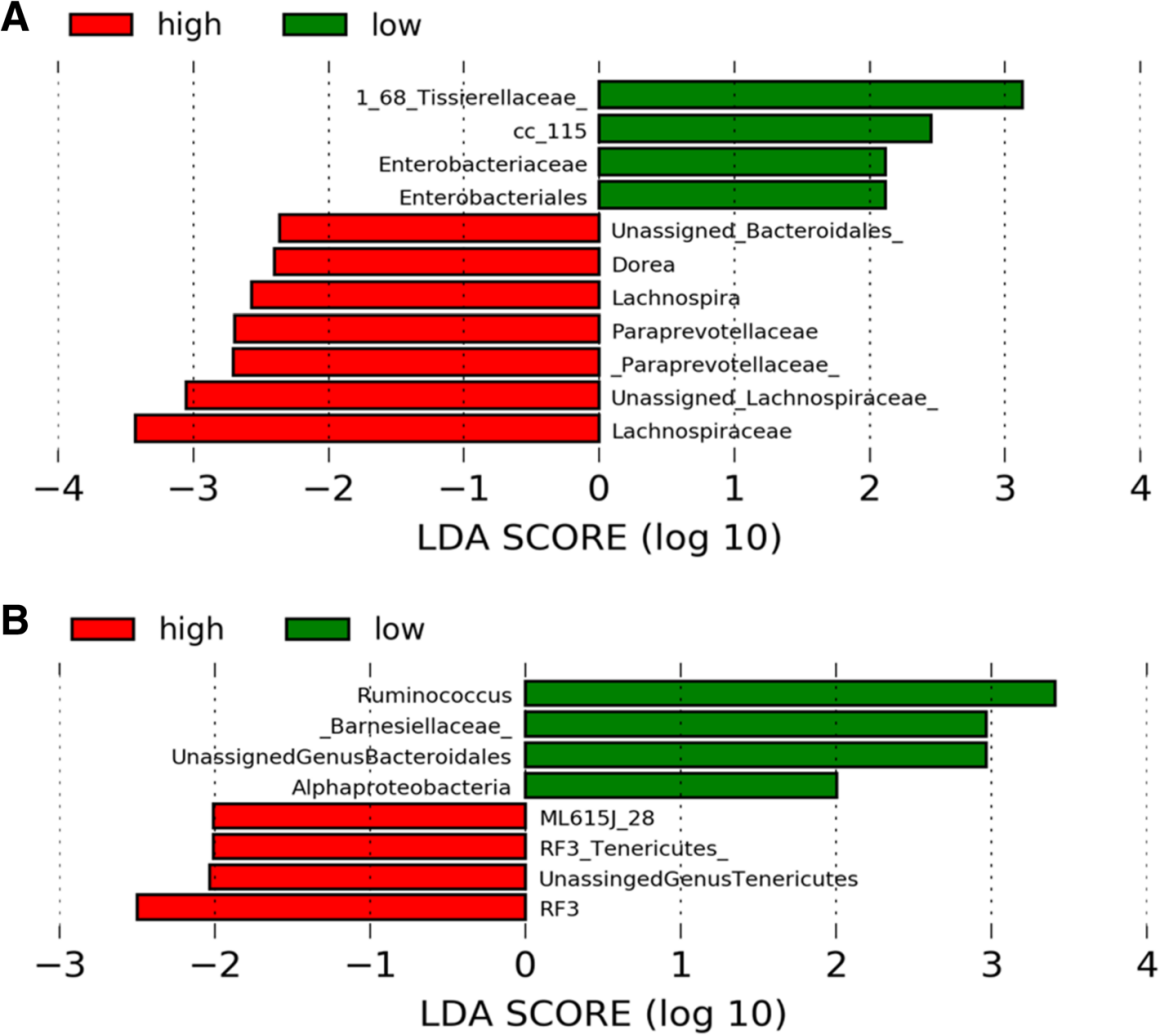
Differential microbial abundance between high and low (**a**) MVPA and (**b**) dietary fiber intake groups. High and low MVPA and fiber groups were greater than or less than median self-reported physical activity (55.7 min/d) and dietary fiber consumption (11.2 g/d), respectively. Identification of differentially abundant microbial taxa was done by linear discriminant analysis effect size analyses (LEfSe). MVPA, moderate-to-vigorous physical activity

Analyses of fecal microbiota in high and low (median cut point) MVPA or dietary fiber groups using LEfSe allowed for the identification of specific bacterial taxa that were associated with self-reported MVPA and dietary fiber consumption. The greatest differences at various taxa levels between the two communities are displayed for MVPA (Fig. 4a) and dietary fiber (Fig. 4b). Data for MVPA suggested significant enrichment of family *Paraprevotellaceae* among those reporting greater MVPA. Family *Lachnospiraceae* and its genus *Lachnospira* were also identified as potential microbial markers of this more active group of college students. Family *Enterobacteriaceae* and genus member *Enterobacteriales* were more enriched among college students reporting MVPA below the median value of 55.7 min/d. The family *Barnesiellaceae*, class *Alphaproteobacteria*, and genera *Ruminococcus* and unassigned *Bacteroidales* were more abundant taxa in the low fiber consumption group, while *Tenericutes* and other unassigned microbes were more abundant among those consuming greater than the median dietary fiber intake.

## Discussion

This study is unique given its focus on college students, a population susceptible to major lifestyle changes that occur during a period of continued social and physical development. In this study, we observed within-group differences in beta-dispersion among a diverse cohort of first-year college students reporting different dietary and physical activity behaviors but these behavioral categories did not explain between-group differences in microbial community structure. Additionally, a merged comparison of dietary fiber consumption and MVPA revealed differences in microbial beta-dispersion such that high MVPA combined with low fiber intake resulted in smaller within-group distances when compared to within-group distances for all other MVPA-fiber groups. Lastly, we report that specific microbial taxa were differentially abundant among college students reporting different daily MVPA and fiber consumption habits.

This study revealed no difference in the F:B ratio by BMI, dietary intake variables, MVPA or screen time categories. Previous literature has highlighted contradictory results with regard to this phyla-level assessment. *Firmicutes* have frequently been shown to decrease with weight loss and have been observed in higher proportions among obese animals and humans when compared to lean counterparts [40–42]. Nonetheless, others have reported no difference in the F:B ratio in relation to BMI or weight change [43–45] or a greater abundance of *Bacteroidetes* among individuals with increased BMIs [46]. *Bacteroidetes* have been positively associated with dietary fat while *Firmicutes* have been associated with dietary fiber [47]. Conversely, a study in children reported greater *Bacteroidetes* among those consuming low-fat, high-starch and fiber diets when compared to children consuming more western-style diets [28]. Still other studies report that diet did not alter the proportions of *Firmicutes* and *Bacteroidetes* [48]. Although such discrepancies also exist among the emerging literature regarding physical activity, exercise and the gut microbiome, these differences in findings may be the result of differing study designs, methodologies, sample types, and lack of replication of data. Similar to our findings, the F:B ratio did not differ among exercise-trained, obesity-prone rats [49] or active adult women [31], when compared to sedentary controls. To date, three studies have suggested a decrease in *Firmicutes* among exercise-trained rodents of varying metabolic status [50–53], while two investigations suggested an increase in *Firmicutes* among exercise-trained rodents [54–56].

Findings from the current study suggest that gut microbial beta-dispersion but not between-group distances differed by categories of high and low macronutrient (carbohydrate, protein and fat) consumption. Carbohydrates, in particular dietary fibers, have been identified as an important fuel source for the gut microbiome as part of habitual diets and short- and long-term dietary interventions [57]. The finding that decreased dietary fiber consumption among college students resulted in beta-dispersion differences may be supported by data suggesting that dietary fiber increases microbial diversity in the gut by altering the composition of fiber-fermenting microbes [28, 58]; however, causality cannot be inferred in this cross-sectional analysis. Previous assessments of lifestyle factors and gut microbiome community structure also support our findings of increased abundance of *Barnesiellaceae*, *Alphaproteobacteria*, and *Ruminococcus*, and decreased *Tenericutes* among low-fiber consuming students compared to high-fiber consumers. *Barnesiellaceae* members have been associated with Western diets limited in fiber [59]. *Ruminococcus* has been associated with the degradation of resistant starches which remain abundant in processed refined grain products [59]; this may explain the observed increase among low-fiber consumers. *Tenericutes* had a greater relative abundance among Bangladeshi children consuming fiber-rich diets compared to American children following Western diets higher in fat and protein and low in fiber [60]. Findings related to dietary fiber in the current study occurred despite the fact that college students in the present study reported consuming a median fiber intake slightly more than 11 g/d. While this falls well below recommended intakes for males and females 38 g/d and 25–26 g/d, respectively [39], this finding suggests that small amounts of dietary fiber may be sufficient to model potentially positive changes in the gut microbiome. Further work is needed to assess specific types of fiber and their influence on specific microbial taxa and evaluate cause and effect relationships in both animals and humans.

Gut microbial taxa varied among students reporting differing levels of daily physical activity with *Paraprevotellaceae*, *Lachnospiraceae,* and *Lachnospira* being more prevalent in college students reporting greater MVPA, and *Enterobacteriaceae* and *Enterobacteriales* being more enriched among college students reporting less MVPA. To date, the majority of evidence for exercise and the gut microbiome is from animal models. A pilot study among elite cyclists also found that *Prevotella* genera abundance was positively correlated with the amount of time spent exercising [61]. Similar to our findings, although in a mouse model with longitudinal measures, Evans et al. reported an increase in *Lachnospiraceae* with voluntary wheel running and an increase in *Ruminococcaeae* but this family may have been equally influenced by the high-fat feeding protocol [50]. In a study of mice with ad-libitum food access, voluntary wheel running resulted in increased fecal *Lactobacillus*, *Bifidobacterium* and *Blautia coccoides–Eubacterium rectale* and decreased *Clostridium* and *Enterococcus* when compared to sedentary controls [53]. Voluntary wheel running among mice has also been shown to revert the negative effects of polychlorinated biphenyl exposure on the gut microbiome with significantly different community structures compared to sedentary mice [51]. Mode of exercise may also play a role in shaping the gut microbiome, as high-intensity interval training 3 times per week for 6 weeks improved the diversity of the colonic gut microbiota in high-fat fed mice [52]. While our study did not capture the types of physical activity in which students were engaging, a recent human study found that endurance exercise-induced changes in the gut microbiota were dependent on body mass suggesting that the metabolic health of individuals should be accounted for in future studies [33]. The majority of participants in the current study were of normal weight and metabolically healthy. We did not observe differences in gut microbiome diversity by BMI in our study (mean ± SD: 24.4 ± 5.5 kg/m^2^, range: 16.9–50.4 kg/m^2^).

While animal models have been helpful for establishing relationships between the gut microbiome, physical activity and exercise, these links have been more difficult in humans. To date, there have been few human studies. A recent study found that the gut microbiome of adult women meeting the World Health Organization recommendations for physical activity differed from that of sedentary women with significantly greater abundance of species associated with metabolic health [31]. Other studies have primarily focused on elite athletes who have a tendency toward extreme dietary and exercise behaviors which make it difficult to extrapolate results to the general public [34, 61]. A study conducted on elite rugby players suggested that the gut microbiome of athletes differed significantly in comparison to healthy weight and obese sedentary controls; however, these community differences also appeared to be influenced by the unique dietary practices of the athletes [34]. In our study, we aimed to assess potential dietary and physical activity interactions by comparing beta-diversity patterns between merged groups of high and low MVPA and fiber. We found that high MVPA combined with low fiber intake resulted in significantly different beta-dispersion than all other combinations of MVPA and fiber consumption but that between-sample distances did not differ by MVPA-fiber categories. This suggests that beta-dispersion patterns might be a marker of specific physical activity and dietary behaviors but inferring causality is not possible given that other unmeasured factors might also be impactful. Kang et al. have reported differential clustering due to exercise on both normal and high-fat diet-fed rats [54] while Welly et al. did not find orthogonal differences in PCoA plots between exercised and dietary-restricted obese rats [49]. Previous work has reported both increases [62] and decreases [51] in fecal *Tenericutes* phylum relative abundance among exercised animals, while our study and work by Lin et al. found that this taxa may differ by fiber-rich foods (legumes, grains) consumption [60]. Work by Kang et al. also suggests that exercise influences *Tenericutes* as this taxa increased despite consumption of standard or high-fat diets [54]. Differential effects on individual gut microbiome taxa require further exploration to better understanding how physical activity and diet independently and mutually influence health.

Limitations of the current study include the small sample size which make it difficult to assess demographic differences in dietary and MVPA behaviors. Further, conclusions regarding gut microbial data should be made with caution as this study was cross-sectional (cannot infer causality) and the collection of a single fecal sample may not accurately capture gut microbiome differences in a free-living population where environmental exposures, diet, physical activity, and other behaviors vary from day to day. Despite not being able to characterize the specific types of physical activity in which college students were engaging at the time of assessment, a strength of this study was the use of validated self-report instruments for assessment of MVPA and dietary intake. Studying a diverse cohort of college students is also a strength as the current microbiome literature has largely ignored this age group and infrequently includes individuals from all races and ethnicities.

## Conclusions

In summary, this study provides observational support for the importance of regular physical activity in shaping the gut microbiome during a period of continued growth and development. Data from this study suggest that while beta-dispersion differed among high and low macronutrient consumers or physical activity categories, between-group distances were not significantly different among these categories. Specific taxa associated with health were differentially more abundant among those reporting greater self-reported fiber intakes and MVPA. While these results are promising, more research is warranted to fully elucidate the role of physical activity and diet in modulating the gut microbiome. Being one of the first studies to examine the gut microbiome in college-aged subjects, opportunities for further investigation include assessment of specific physical activities, exercise interventions assessing different modes and duration of activity, and evaluation of diet and physical activity interactions. Next steps will include hypothesis testing in suitable animal models and human cohorts that utilize carefully designed, longitudinal approaches to elucidate cause and effect relationships between dietary and physical activity effects on the gut microbiome. This work will further identify microbial biomarkers of health and enhance our understanding of how changes in diet and physical activity impact health outcomes including weight gain, a common health outcome among college students and humans of all ages.

## Materials and methods

Healthy college students living in on-campus housing, who were English speaking, and at least 18 years of age were eligible to participate in this cross-sectional study. This cohort of eligible students were recruited from a larger study [63] that used mobile ecological momentary assessment methodology to assess the influence that social networks have on physical activity, dietary intake, and body weight in two residence halls at Arizona State University in Tempe, Arizona. Exclusion criteria for this study included a history of malabsorptive disorders, high blood pressure, eating disorders, HIV infection, diabetes, and/or the use of antibiotics, antifungals, or probiotics in the 2 to 3 months prior to the study. This study was conducted during the Fall 2014 and Spring 2015 semesters. The Arizona State University (ASU) Institutional Review Board approved (STUDY00002019) this study and all participants provided written informed consent.

Participant age, gender, race/ethnicity, and other demographic data were provided via a self-reported, web-based questionnaire that was completed upon entry into the parent study. Height and weight measurements were measured by trained research staff. Each measurement was taken up to three times and the two closest values within 0.5 cm and 0.5 kg of each other, respectively, were averaged. These averaged values were then used to calculate body mass index (BMI) and categorize participants based on the CDC guidelines as follows: BMI < 18.5 kg/m^2^ was considered underweight; BMI ≥ 18.5 kg/m^2^ and ≤ 24.9 kg/m^2^ was considered normal weight; BMI ≥ 25.0 kg/m^2^ and ≤ 29.9 kg/m^2^ was considered overweight; and BMI ≥ 30.0 kg/m^2^ was considered obese [64].

Physical activity habits were determined using the Godin-Shephard Leisure-Time Physical Activity Questionnaire (see Additional file 2) [65]. The Godin-Shephard protocol has been validated as an appropriate method to measure physical activity habits in college-aged males and females [65]. Sedentary activities were also measured using a validated survey (Additional file 2) [66].

The ASA24 24-h dietary recall was used to assess students’ habitual dietary intake. Food and beverage intake was recorded from midnight to midnight on the previous day. The website provided images to guide participants on selecting the correct portion size for each item they consumed. Participants were asked to complete 3 days of dietary recall (2 weekdays and 1 weekend day) which has been validated as a representative and accurate summary of habitual nutrient intake [67, 68]. Days of intake were dropped if caloric intake was below 500 or in excess of 5000 kcal. If a participant did not have at least 1 day of adequate dietary intake they were excluded from the study. The validated ASA24 [69] utilizes the US Department of Agriculture’s Automated Multiple Pass Method (AMPM) [70] and measures intake by using the USDA’s Food and Nutrient Database for Dietary Studies (FNDDS). Using data from the ASA24–2014 Daily Total Nutrients Analysis File (TN), we examined total grams of protein, fat, carbohydrates, and fiber.

Each study participant was provided with a fecal sample collection kit (Commode Specimen Collection Kit, Fisher Scientific, Anthem, AZ) in order to provide a single fecal sample for analysis. Collection kits were distributed to participants in small insulated cooler bags containing ice packs to keep samples cold while in transit post-collection. Before participants left with the kit, a brief demonstration on how to collect the sample was provided along with a sheet of instructions inside the cooler bag. Participants were asked to freeze their ice packs immediately so that they were frozen at the time of sample collection. Ice packs were rated to stay frozen for 36–48 h in an insulated container. All stool samples were retrieved from participants and delivered to the clinical research facility within 24 h of collection. Stool samples were stored at − 80 °C to preserve the microbial community.

Assessment of the gut microbiome in fecal collections was carried out at the Biodesign Institute at ASU in Tempe, Arizona. Extraction of microbial DNA from fecal samples was accomplished using the PowerSoil DNA isolation kit as described by the manufacturer (MoBio Laboratories Ltd., Carlsbad, CA) using a beadbeater (BioSpec, Bartlesville, OK). Amplification of the 16S rRNA gene sequence was completed in triplicate PCRs using 96-well plates. Barcoded universal forward 515F primers and 806R reverse primers containing Illumina adapter sequences, which target the highly conserved V4 region, were used to amplify microbial DNA [71, 72]. These primers were selected as they are recommended by the Earth Microbiome Project [71, 72] and the National Institutes of Health Human Microbiome Project [73] to enhance reproducibility and comparability to other studies while obtaining broad coverage of *Bacteria*. PCR, amplicon cleaning and quantification were performed as previously outlined [72]. Equimolar ratios of amplicons from individual samples were pooled together before sequencing on the Illumina platform (Illumina MiSeq instrument, Illumina, Inc., San Diego, CA) at ASU’s DNASU Genomics Core Facility. Raw Illumina microbial data were cleaned by removing short and long sequences, sequences with primer mismatches, uncorrectable barcodes, and ambiguous bases using the Quantitative Insights Into Microbial Ecology (QIIME) software, version 1.9.1, as previously described [74]. Taxonomic assignments and operational taxonomic units (OTUs) were determined using the closed reference Greengenes database [75] at 99% similarity. The OTU table was filtered for singletons by using the QIIME script filter_otus_from_otu_table.py. OTUs observed fewer than two times were removed from the table.

All statistical analyses were completed using JMP Pro 13 and QIIME 1.9.1 statistical and bioinformatics software packages. Data were expressed as mean ± SD or median (interquartile range) of microbiota frequencies/proportions based on the normality of the data. BMI data were expressed both continuously and categorically (underweight, normal weight, overweight, and obese). Wilcoxon-Kruskal Wallis tests were carried out to assess group differences (e.g. BMI, screen time, MVPA) in the *Firmicutes:Bacteroidetes* (F:B) ratio. Phylogenetic diversity measures were carried out in QIIME to determine alpha (within-sample) diversity metrics via Faith’s PD. Principal coordinates analysis (PCoA) was performed for beta (between-sample) diversity analysis, using both weighted and unweighted unique fraction metric (UniFrac) distances (measure of phylogenetic distance between sets of taxa in a phylogenetic tree as a fraction of the branch length on the tree), on the 99% OTU composition and abundance matrix [76]. UniFrac distance metrics group comparisons were performed for self-reported MVPA, screen time, and dietary intake variables. Linear discriminant analysis (LDA) effect size (LEfSe) was performed to identify microbial taxa that were differentially abundant by MVPA and dietary fiber consumption groups [77]. Findings were considered significant at *p* < 0.05 following adjustments for multiple comparisons. Sample sequences were deposited at the NCBI/Sequence Read Archive (SRA) under project PRJNA473006 with accession numbers: SAMN09258197 - SAMN09258278.

## Additional files

Additional file 1: File showing PCoA plots for all dietary intake, physical activity, and screen time group median or quartile comparisons. (PDF 1045 kb)

Additional file 2: Physical Activity and Sedentary Activity Questionnaire Details. This file offers additional information regarding questions used in survey instruments and scoring methods to tabulate or summarize data for statistical analyses. (PDF 69 kb)

Additional file 3: Table showing participant characteristics including demographics, behavioral data and alpha diversity metrics. (XLSX 21 kb)

Additional file 4: Table showing taxonomic abundance data for all genera. (XLSX 113 kb)

## Abbreviations

AMPM: US Department of Agriculture’s Automated Multiple Pass Method
BMI: Body mass index
F:B: Firmicutes:Bacteroidetes
FNDDS: USDA’s Food and Nutrient Database for Dietary Studies
LDA: Linear discriminant analysis
LEfSe: Linear discriminant analysis (LDA) effect size
MVPA: Moderate-to-vigorous physical activity
OTU: Operational taxonomic unit
PCoA: Principal coordinates analysis
UniFrac: Unique fraction metric
