# Supplementary material for: Diet, physical activity and screen time but not body mass index are associated with the gut microbiome of a diverse cohort of college students living in university housing: a cross-sectional study
Source: BMC Microbiol. 2018 Dec 12;18:210. doi: 10.1186/s12866-018-1362-x (PMC6291939; doi:10.1186/s12866-018-1362-x)
Supplement: Supplementary file 1 — File showing PCoA plots for all dietary intake, physical activity, and screen time group median or quartile comparisons. (PDF 1045 kb) [file 12866_2018_1362_MOESM1_ESM.pdf]

Moderate-to-Vigorous Physical Activity (min/d)

Unweighted UniFrac

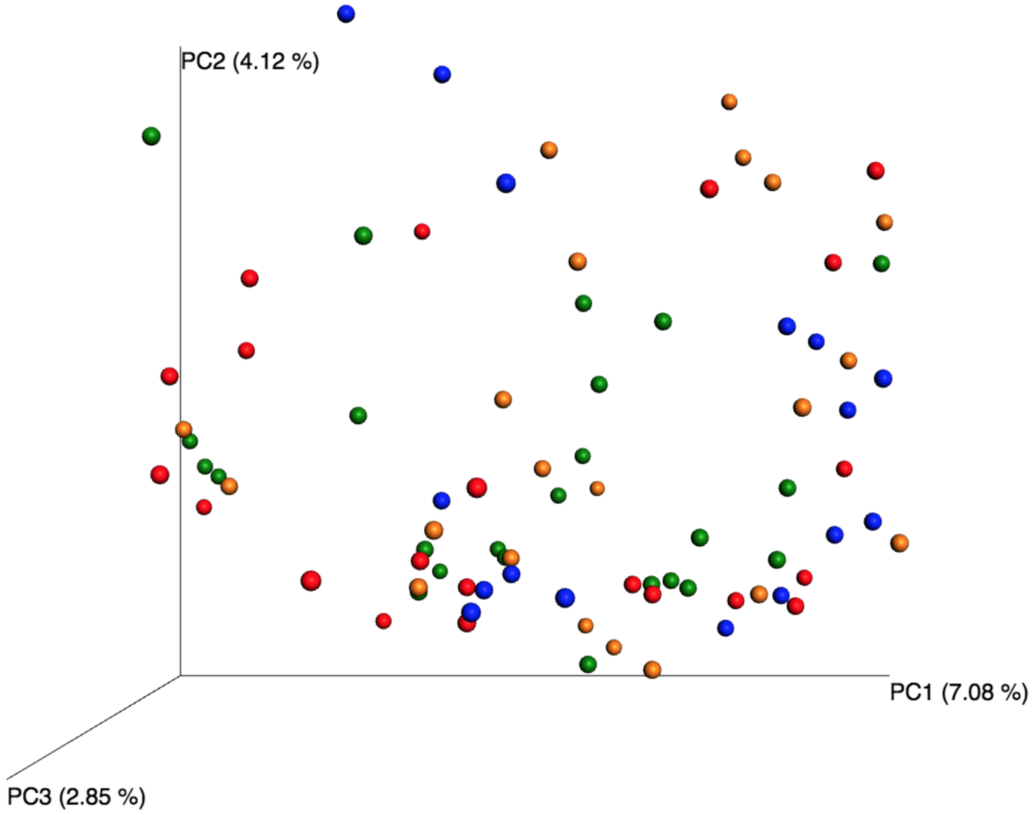

Weighted UniFrac

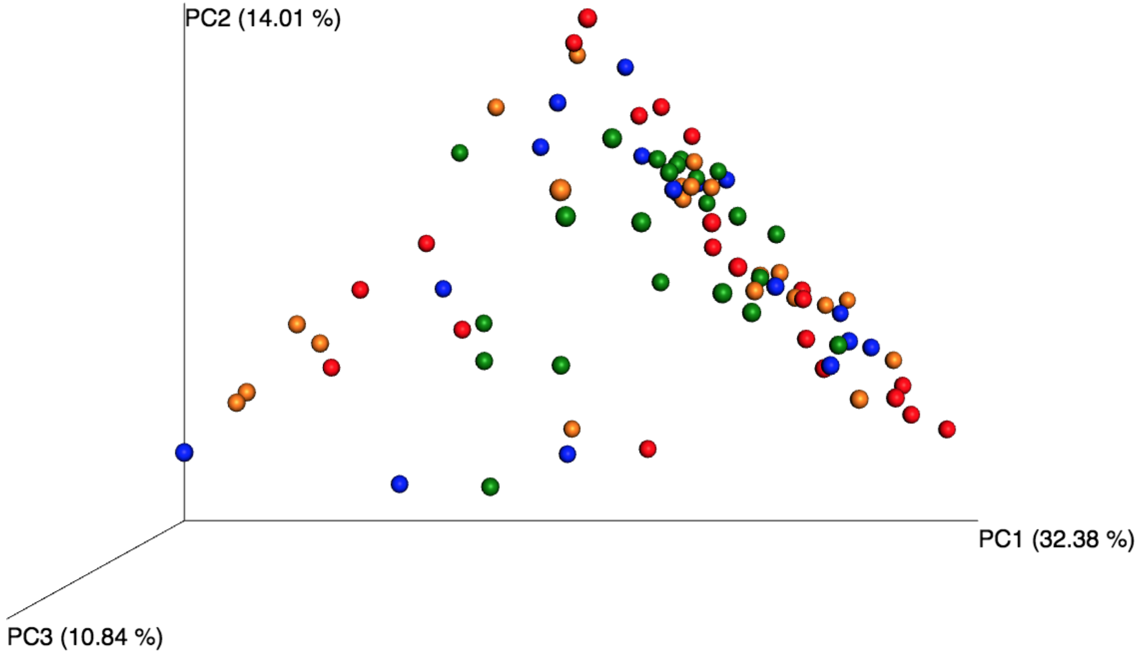

< 28.8   28.8-55.7   55.8-79.2   79.3-102.9

Screen time (hrs/d)

Unweighted UniFrac

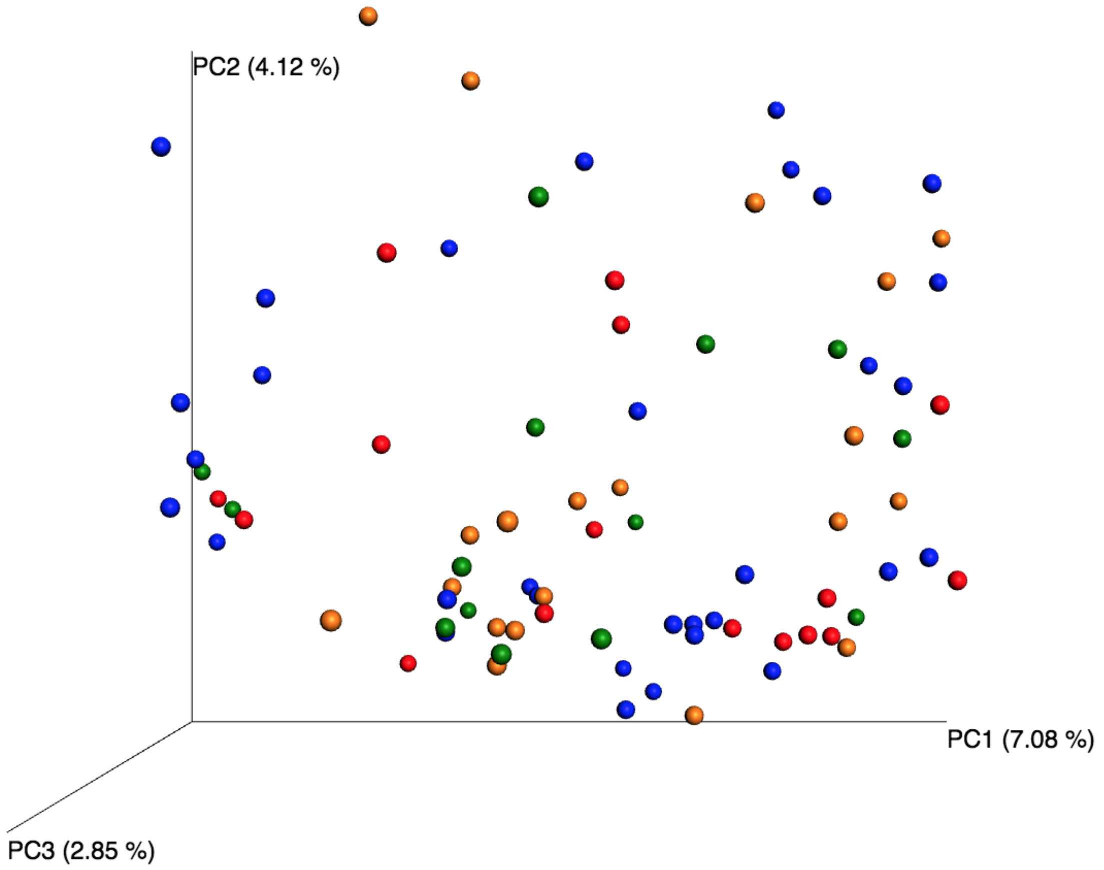

Weighted UniFrac

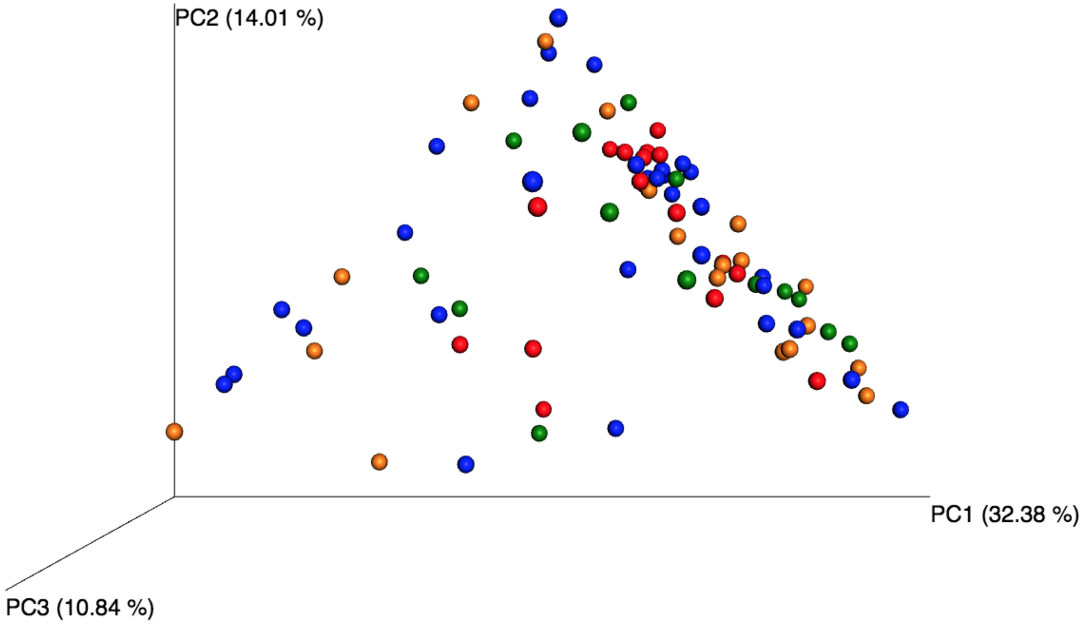

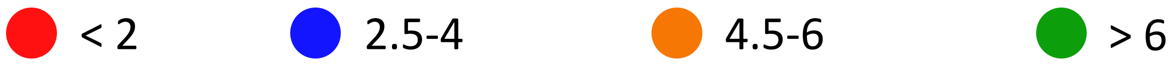

Dietary Protein Intake (g/d)

Unweighted UniFrac

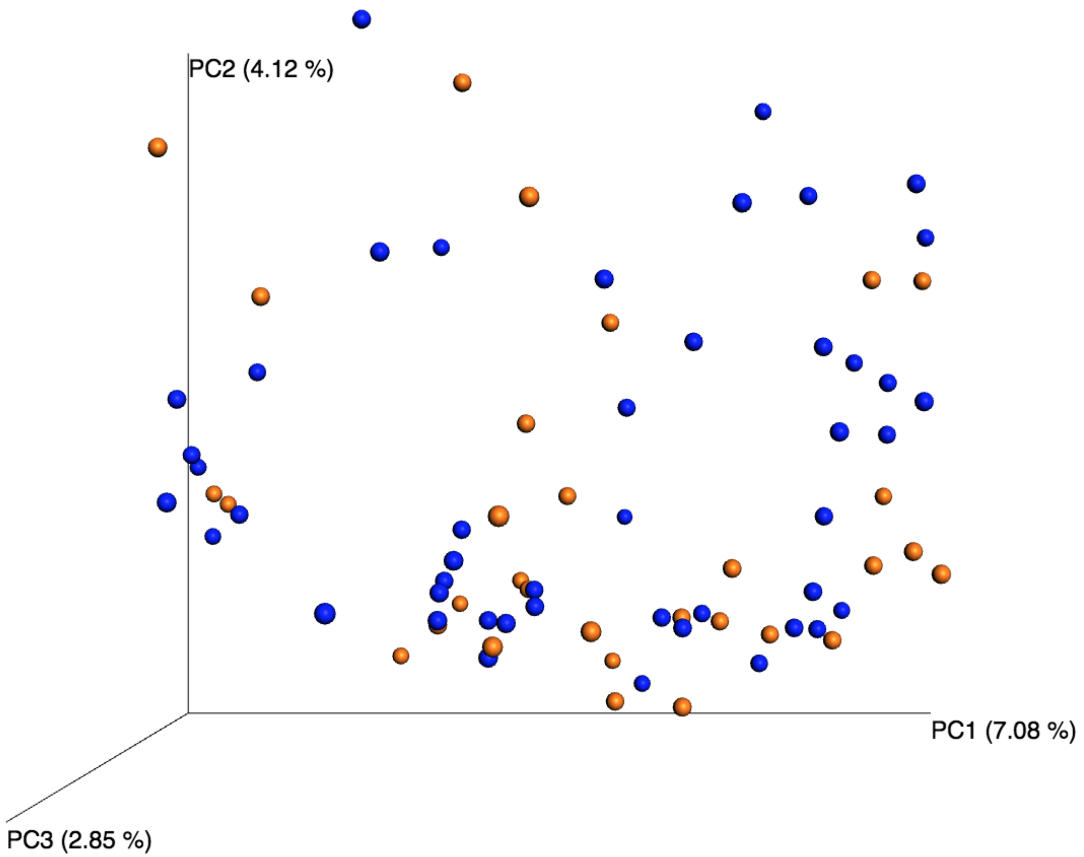

Weighted UniFrac

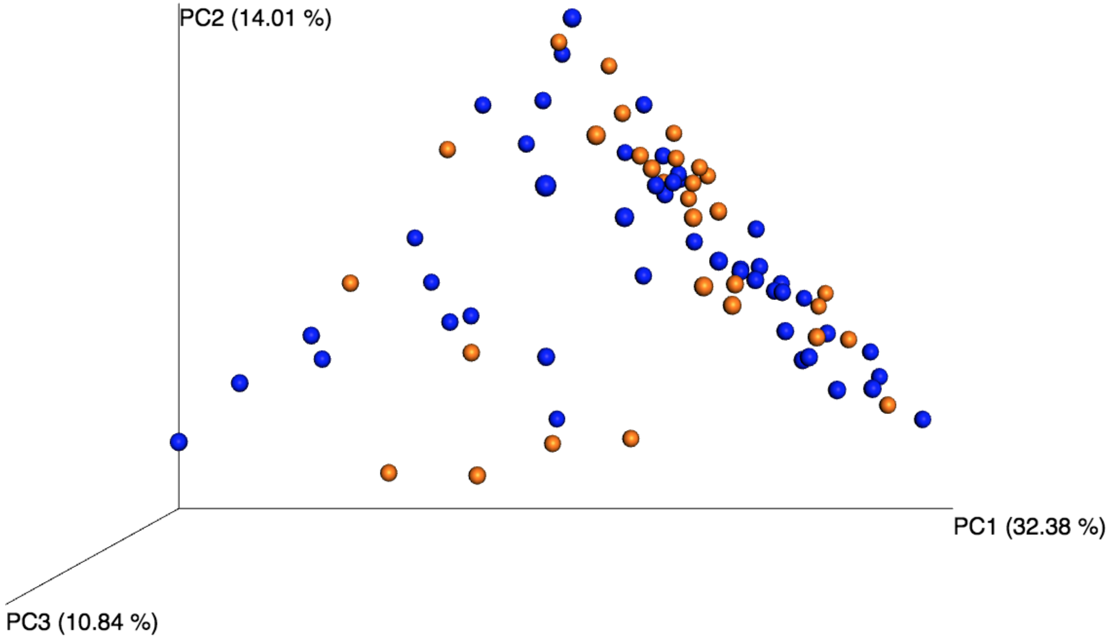

● < 62 g/d ● > 62 g/d

Dietary Fat Intake (g/d)

Unweighted UniFrac

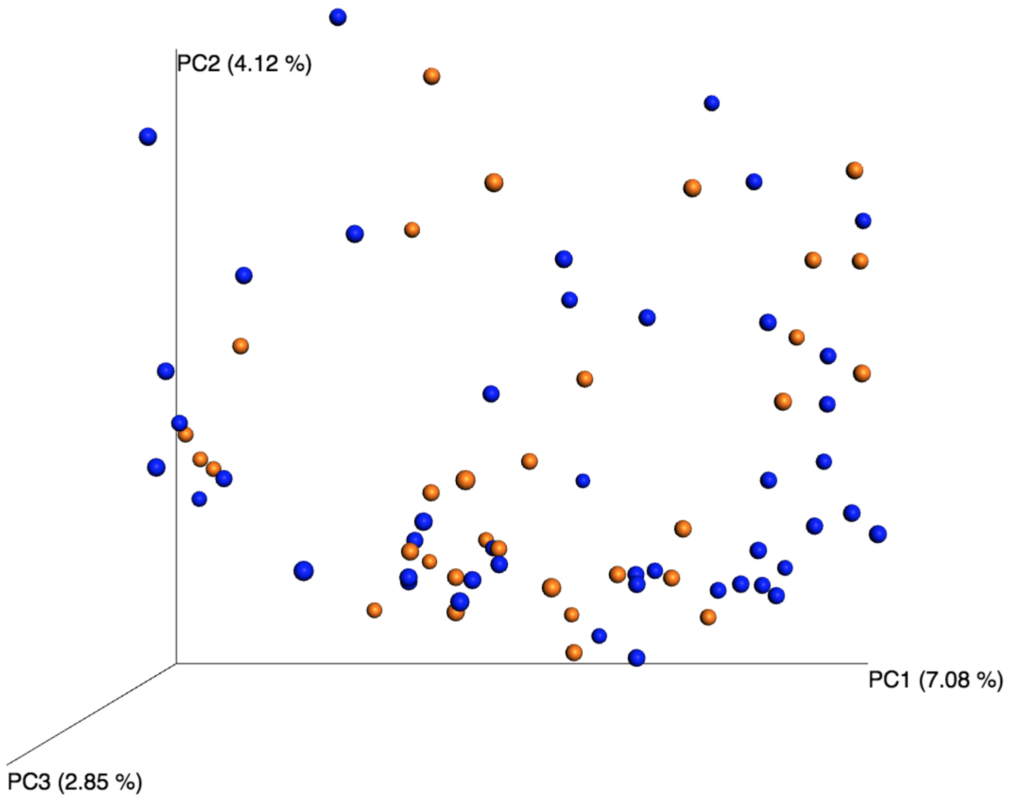

Weighted UniFrac

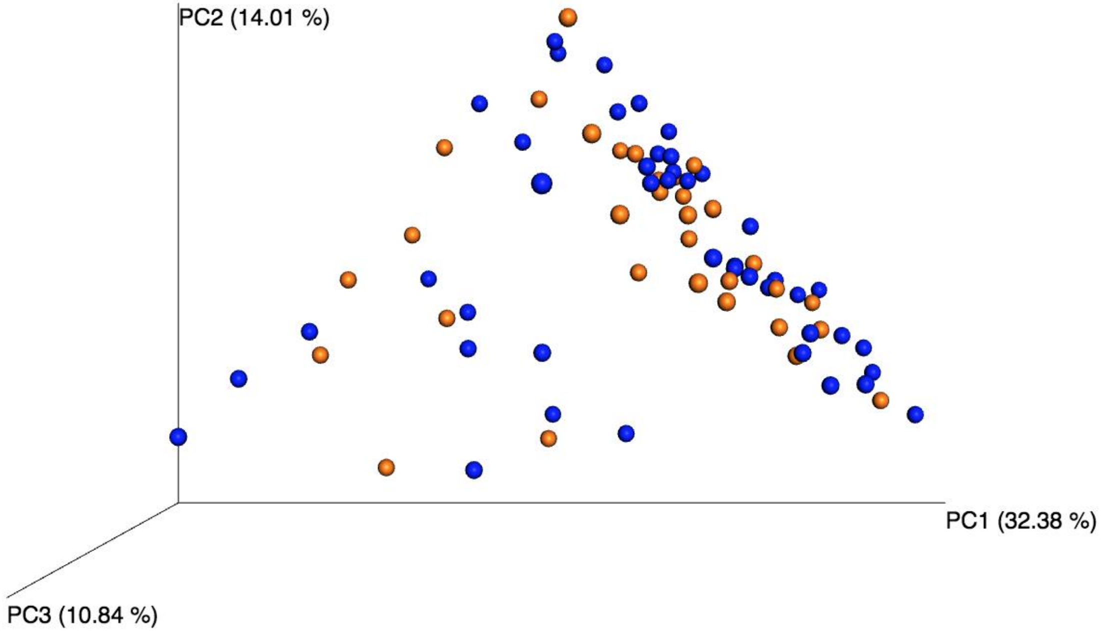

< 63 g/d   > 63 g/d

Dietary Carbohydrate Intake (g/d)

Unweighted UniFrac

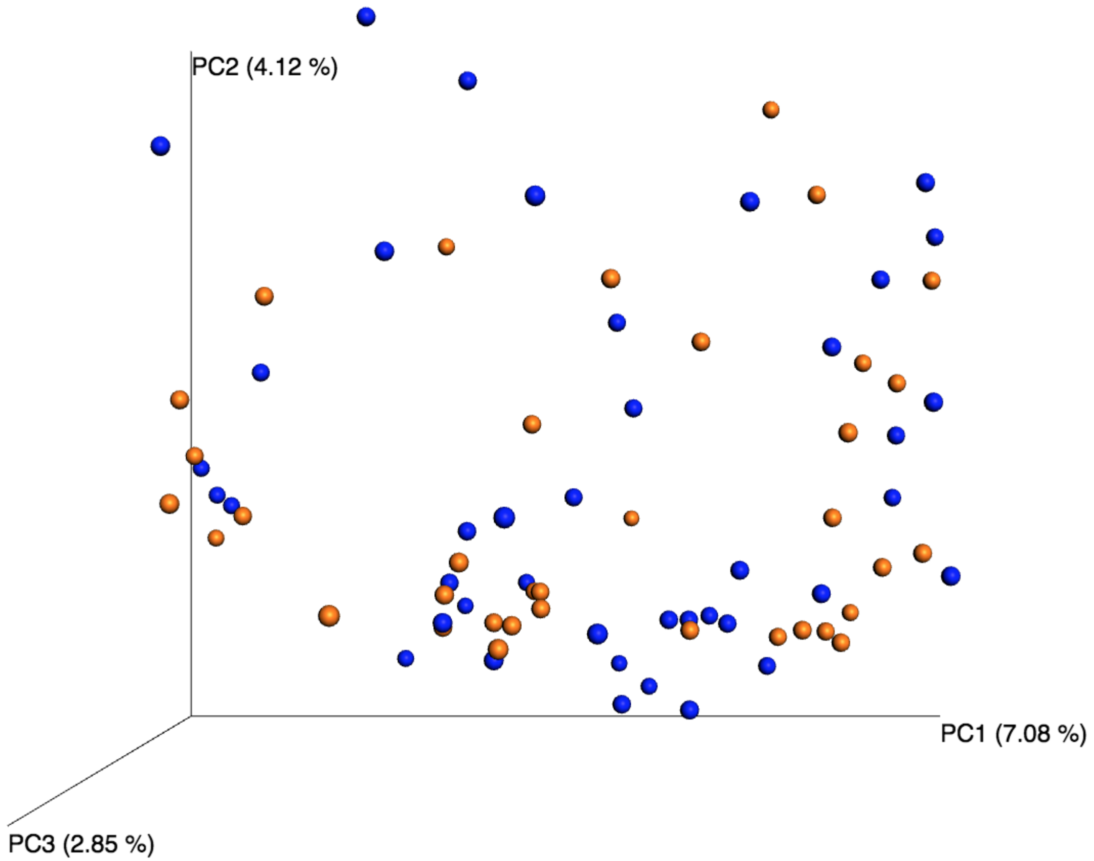

Weighted UniFrac

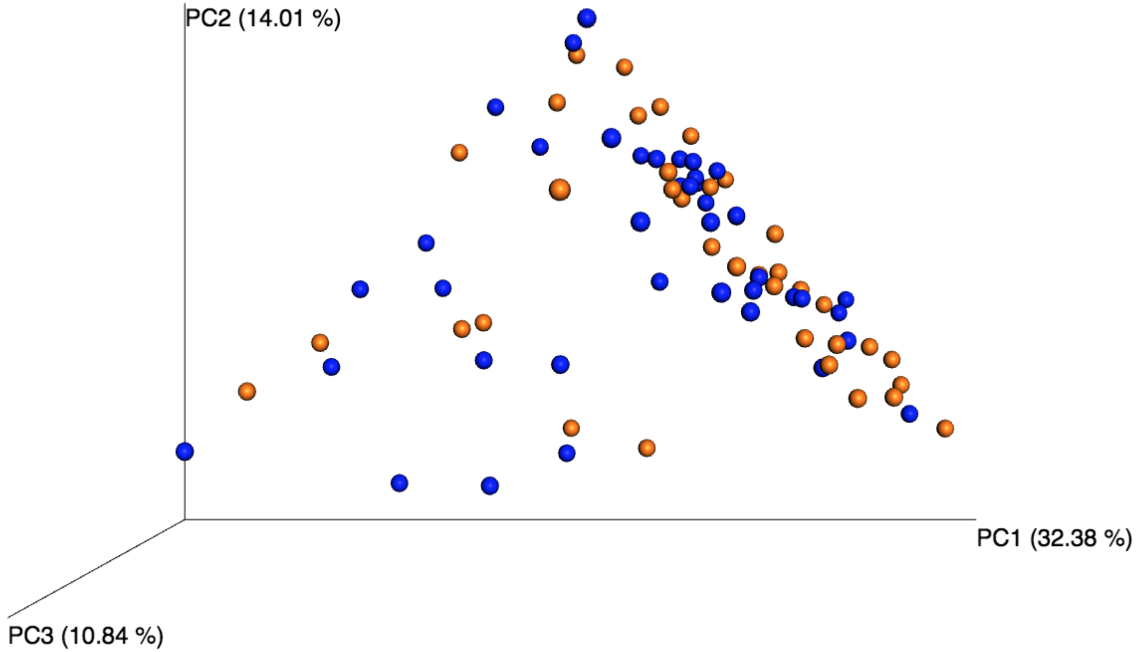

● < 166 g/d ● > 166 g/d

Dietary Fiber Intake (g/d)

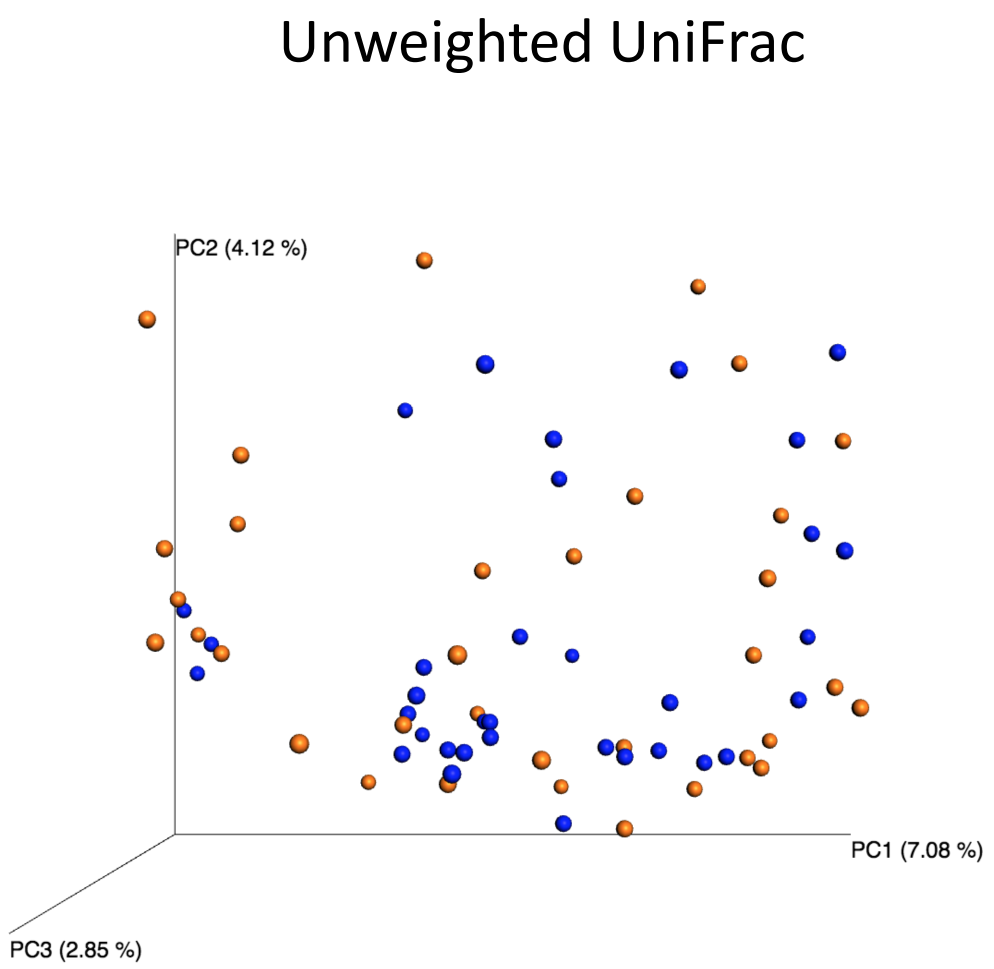

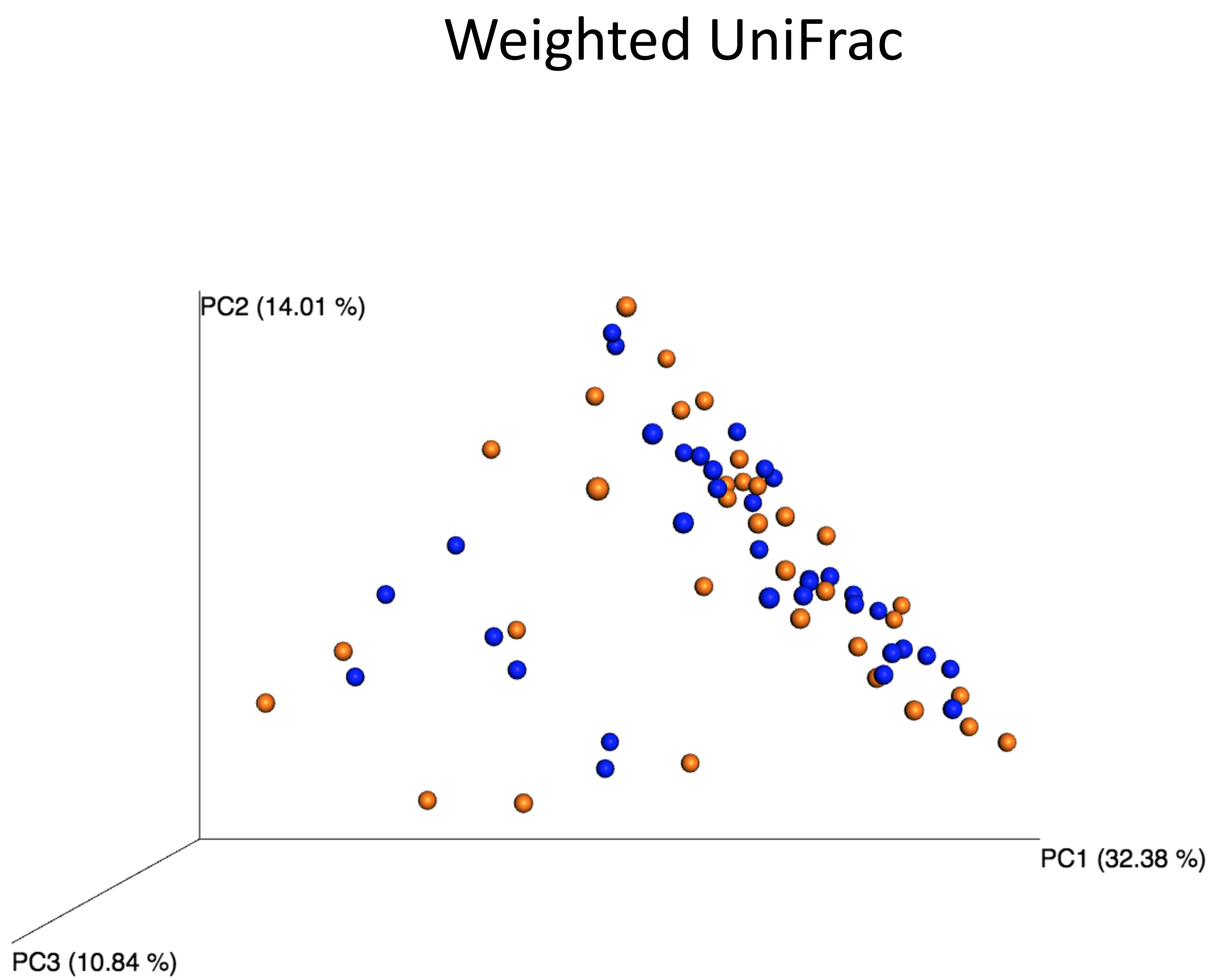

< 11 g/d   > 11 g/d

Physical Activity and Fiber Mixed Categories

Unweighted UniFrac

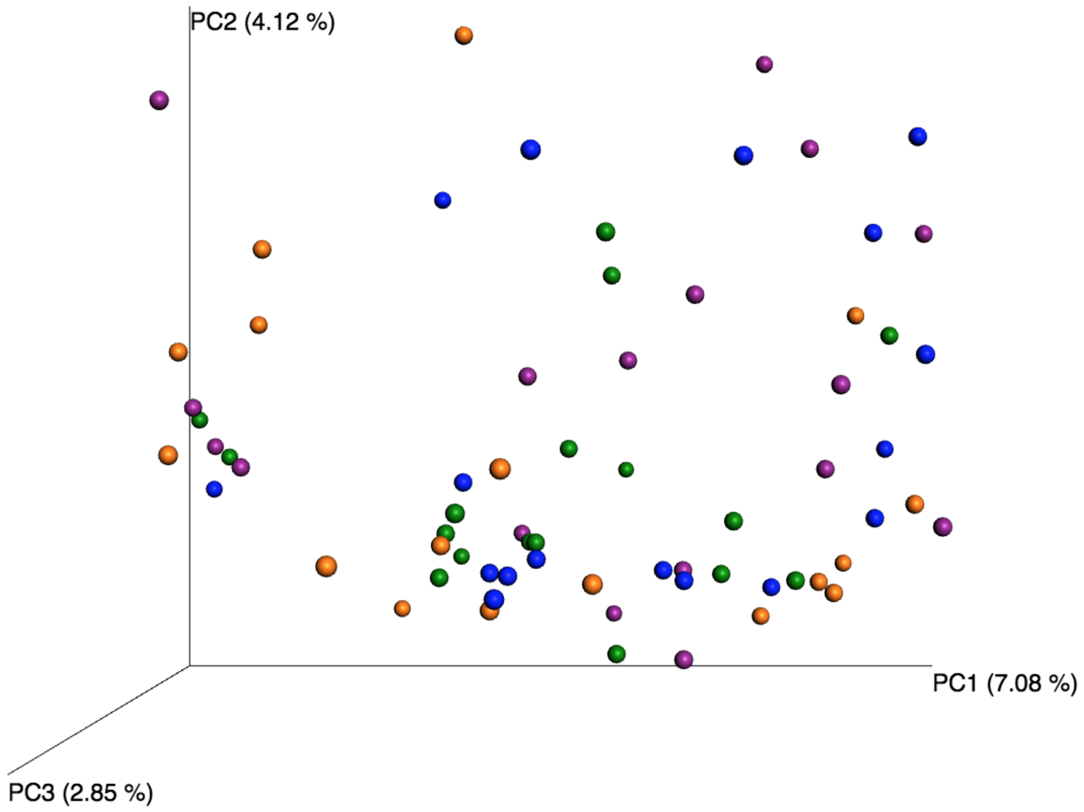

Weighted UniFrac

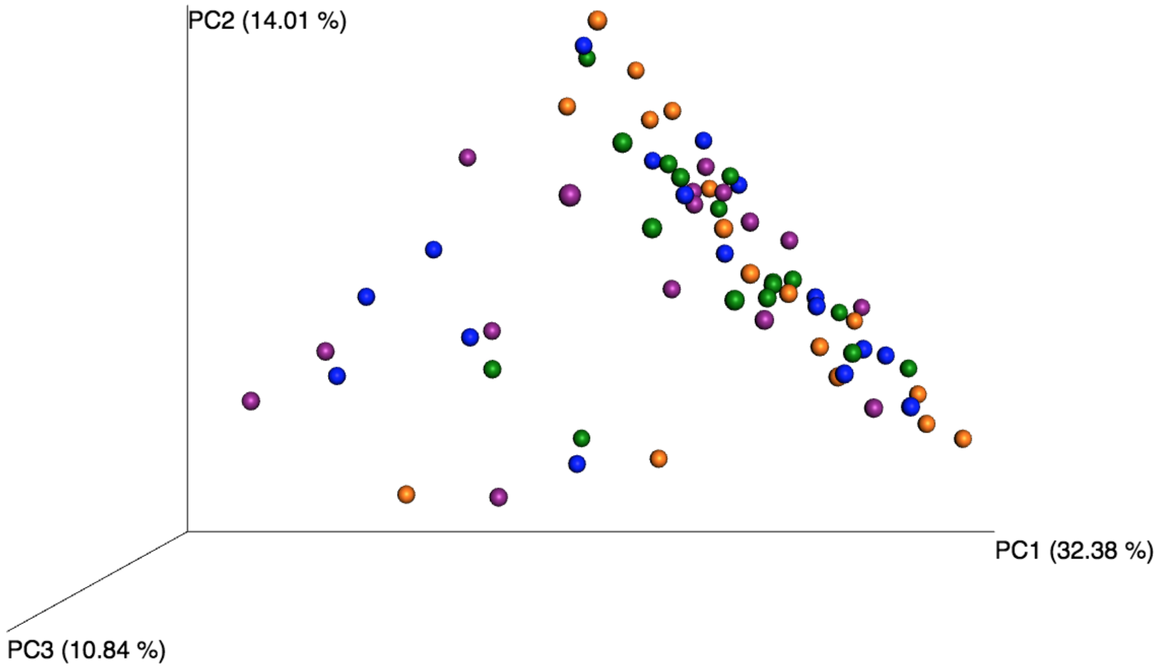

MVPA – moderate-to-vigorous physical activity

Low MVPA-Low Fiber   Low MVPA-High Fiber   High MVPA-Low Fiber   High MVPA-High Fiber
