# Supplementary material for: Diet, physical activity and screen time but not body mass index are associated with the gut microbiome of a diverse cohort of college students living in university housing: a cross-sectional study
Source: BMC Microbiol. 2018 Dec 12;18:210. doi: 10.1186/s12866-018-1362-x (PMC6291939; doi:10.1186/s12866-018-1362-x)
Supplement: Supplementary file 2 — Physical Activity and Sedentary Activity Questionnaire Details. This file offers additional information regarding questions used in survey instruments and scoring methods to tabulate or summarize data for statistical analyses. (PDF 69 kb) [file 12866_2018_1362_MOESM2_ESM.pdf]

**Additional File 2. Physical Activity and Sedentary Activity Questionnaire Details**

**Godin-Shephard Leisure-Time Physical Activity:** The questionnaire asked students, "In a usual week, how many hours do you spend doing the following activities: strenuous physical activity (heart beats rapidly), moderate physical activity (not exhausting), and mild physical activity (little effort)." The response options were: none, less than ½ hour per week, ½-2 hours per week, 2½-4 hours per week, 4½-6 hours per week, and more than 6 hours per week. Daily physical activity was calculated and moderate-to-vigorous physical activity (MVPA) was computed by summing the total time spent on strenuous and moderate activity.

**Sedentary Activity Question:** Sedentary activities were also measured by asking: "Yesterday, how much time did you spend in front of a screen (excluding time in class and being physically active)?" Response options included a range from zero to greater than 6 hours which were summed to calculate total time spent doing sedentary activities.
